# Association of paternal MTHFR polymorphisms (C677T) with clinical outcomes in ICSI treatment

**DOI:** 10.3389/fendo.2022.1084463

**Published:** 2022-12-22

**Authors:** Yangyang Wan, Wenjing Liu, Bo Xu, Xiaohua Jiang, Juan Hua

**Affiliations:** ^1^ Reproductive and Genetic Hospital, The First Affiliated Hospital of USTC, Division of Life Sciences and Medicine, University of Science and Technology of China, Hefei, China; ^2^ Research Center for Translational Medicine, The Second Hospital of Anhui Medical University, Hefei, China; ^3^ Department of Biochemistry and Molecular Biology, School of Basic Medical Sciences, Anhui Medical University, Hefei, China

**Keywords:** MTHFR (C677T), intracytoplasmic sperm injection (ICSI), sperm DFI, male infertility, assisted reproductive technologies

## Abstract

**Purpose:**

This study aims to investigate the association between paternal methylenetetrahydrofolate reductase (MTHFR) polymorphisms (C677T) and embryonic development, pregnancy, and neonatal outcomes in intracytoplasmic sperm injection (ICSI) treatment.

**Methods:**

A total of 191 infertile men undergoing ICSI treatment at the Reproductive and Genetic Hospital, The First Affiliated Hospital of USTC, were recruited between January 2020 and June 2021. The MTHFR C677T polymorphism genotyping was evaluated in these male patients, and they were stratified into three groups according to genotyping results: Control (CC), heterozygote mutated (CT), and mutated homozygote (TT). In addition, we conducted a comparative analysis of embryonic development, pregnancy, and neonatal outcomes among these three groups.

**Results:**

The embryonic development (including normal fertilization rate (80.14% vs. 83.06% vs. 85.10%; *p* = 0.37), high-quality embryo rate (45.26% vs. 43.69% vs. 46.04%; *p* = 0.72), blastocyst formation rate (42.47% vs. 43.18% vs. 39.38%; *p* = 0.62), implantation rate (42.47% vs. 36.25% vs. 41.22%; *p* = 0.62), and clinical pregnancy rate (64.71% vs. 58.75% vs. 66.67%; *p* = 0.59) were not comparable among these three groups. Moreover, no significant difference was observed in terms of pregnancy outcomes (including miscarriage rate (24.24% vs. 12.77% vs. 22.5%; *p* = 0.35) and live birth rate (49.02% vs. 51.25% vs. 51.66%; *p* = 0.96)). Additionally, no marked difference was observed in terms of neonatal outcome (including, preterm delivery rate (24% vs. 14.63% vs. 9.67%; *p* = 0.35), birth height (*p* = 0.75), birth weight (*p* = 0.35), neonatal sex (*p* = 0.48), gestational age at delivery (*p* = 0.24), Apgar score (*p* = 0.34), and birth defects (0% vs. 2% vs. 9%; *p* = 0.23) among the study groups.

**Conclusion:**

The paternal MTHFR C677T polymorphism is not associated with embryo quality, pregnancy, or neonatal outcomes in ICSI treatment. Therefore, in our population, MTHFR polymorphisms do not provide helpful information in explaining ICSI failure.

## Introduction

Assisted reproductive technologies (ART) have become widely accepted as a proven and routine treatment for infertility (1). However, despite recent advancements in *in vitro* fertilization (IVF) and intracytoplasmic sperm injection (ICSI), only about one-third of these procedures result in live births (2). Understanding the factors that affect the outcome of IVF/ICSI pregnancies is crucial to designing safer and more effective infertility treatments (2). ICSI procedure failures are likely caused by several factors, including the woman’s age, sperm and oocyte quality, as well as poor uterine receptivity (3). Previous literatures have suggested that abnormal semen parameters, chromosomal anomalies, sperm DNA fragmentation index (DFI), and genetic–epigenetic aberrations adversely affect pregnancy outcomes following ICSI procedures (4–6). According to a number of studies, methylenetetrahydrofolate reductase (MTHFR) polymorphisms are associated with oxidative stress, sperm parameters, and sperm DFI (7), implying that MTHFR polymorphisms may serve as a genetic risk factor for ICSI.

The human MTHFR gene is located on the short arm of chromosome 1 and encodes one of the regulatory enzymes controlling folate metabolism (8, 9). MTHFR catalyzes 5,10-methylenetetrahydrofolate to turn into 5-methylenetetrahydrofolate (5-MTHF), which is necessary for homocysteine’s (Hcy) conversion into methionine *via* the methionine synthesis pathway (10). There are three commonly known polymorphisms of the MTHFR gene, namely MTHFR C677T (rs1801133), MTHFR A1298C (rs1801131), and MTHFR G1793A (rs2274976) (11). The MTHFR C677T polymorphism alters an alanine (Ala) to a valine (Val), which decreases the thermal stability of the enzyme (8). The enzyme activity in homozygous TT mutant individuals is around 30% of that in homozygous CC genotype individuals, whereas enzyme activity in heterozygous genotype (CT) individuals is approximately 65% of that in homozygous CC genotype individuals (12). The MTHFR polymorphism (C667T) was significantly associated with a higher risk of male infertility in the Chinese population, while the MTHFR polymorphism (A1298C) was not considered a risk factor for male infertility, according to numerous studies (8, 13, 14). Some studies have also examined the association between MTHFR polymorphisms, recurrent spontaneous abortions, and recurrent implantation failures (15–17). Although most current reports have focused on women-related factors (18–20), sperm quality may also play an important function. As a result, it is necessary to investigate the correlation between the paternal MTHFR C677T polymorphism and the clinical outcomes of ICSI treatment.

## Materials and methods

### Study population

In this study, 191 patients undergoing ICSI treatment at the Reproductive and Genetic Hospital, The First Affiliated Hospital of USTC, were recruited between January 2020 and June 2021. Male participants were enrolled from infertile couples due to a male factor.

There were no initial exclusion criteria. As part of their initial prenatal counseling visit, all females were advised to take a prenatal vitamin containing at least 400 micrograms of folic acid.

### Semen analysis

Semen samples from the male patients were obtained after 2–7 days of abstinence and assessed after liquefaction at 37°C for 30 min. Semen parameters were determined according to the World Health Organization (WHO) guidelines for semen analysis (fifth edition, 2010). Sperm concentration, progressive motility, and total motility were assessed by computer-assisted sperm analysis (CASA) under a phase-contrast microscope (CX43, Olympus Corporation, Tokyo, Japan) equipped with a SAS-II system (SAS Medical, Beijing, China). Sperm morphology was evaluated through Diff-Quick staining (Ankebio, Hefei, China) at ×100 magnification under a light microscope (UB100i, UOP, Chongqing, China). Leukocytes were stained with benzidine for the peroxidase test (Ankebio). Antisperm antibody (AsA) levels were measured by the mixed antiglobulin reaction (MAR) method (Ankebio). Sperm DNA fragmentation was evaluated by flow cytometry according to the manufacturer’s protocol (Cellpro, Ningbo, China).

### Genotype detection

The whole genome DNA was extracted using QIAquick PCR purification kits (QIAGEN, Germany) from the blood samples of the male patients. The MTHFR C677T genotype was analyzed using a fluorescence PCR detection kit (PCR-fluorescence probe) produced by Tailored Medical (Shenzhen, Guangdong, China). Fluorescence PCR detection was conducted under the following conditions: a 4-μl whole genome DNA and a 10-μl PCR reaction system. Next, the manufacturer recommends 95°C denaturation for 15 s and 60°C annealing/extension for 60 s, with 45-cycle reaction conditions. After the PCR reaction, the endpoint fluorescence in each sample well was measured using the ABI 7500 fluorescence quantitative PCR instrument (Applied Biosystems, Foster City, CA, USA), and the MTHFR genotyping results were accurately determined using the ABI 3730 Genetic Analyzer (Applied Biosystems, Foster City, CA, USA).

### Intracytoplasmic sperm injection

ICSI was conducted as previously described (21). The women were subjected to controlled ovarian hyperstimulation using a standard long protocol. First, oocyte retrieval was performed 36 h after 250 µg recombinant hCG (Ovitrelle, Merck Serono, Switzerland) administration. Next, oocytes were treated with a G-morpholinepropanesulfonic acid (MOPS) medium (Vitrolife, Kungsbacka, Sweden). The embryos were then denuded with hyaluronidase using G-*in vitro* fertilization (IVF) PLUS medium (Vitrolife). Embryos were cultured in a G-1 medium (Vitrolife) in a desktop incubator (COOK Medical, Bloomington, IN, USA) for at least 3 days. Progesterone was used for luteal support.

### Determination of pregnancy

Clinical pregnancy was confirmed *via* two blood hCG tests conducted 14 days after transplantation. An ultrasound screening was performed 30 days after transplantation to detect visible sacs and to evaluate their development.

### Statistical analysis

Quantitative variables of normal distribution were represented using mean ± standard deviation (SD). If not, medians (interquartile range (IQR)) are used. Qualitative variables were described as frequency and percentage; for comparative analysis among the three groups, analysis of variance (ANOVA) was employed for quantitative variables with normal distribution, and the Kruskal–Wallis test was used for the nonnormal distribution. Pearson’s chi-square test or Fisher’s exact test was employed for qualitative variables. Observed frequencies of different genotypes were separately tested for deviation from the Hardy–Weinberg equilibrium using the exact test. All significance tests were two-sided, and a *p* < 0.05 was regarded as statistically significant. Statistical analyses were performed using R (version 3.5.3).

## Result

### Baseline characteristics

A total of 191 men from infertile couples were recruited for this study. MTHFR genotyping analysis for locus 677 was conducted in these patients. In total, we observed homozygosity for the C allele in 26.70% (*n* = 51), heterozygosity in 41.88% (*n* = 80), and homozygosity for the T allele in 31.42% (*n* = 60) of patients. According to published studies, our infertile male patients had a greater frequency of the MTHFR 677T allele than the general population, and the allele frequency for C677T was in Hardy–Weinberg equilibrium (**Table 1**).

**Table 1:** Genotype frequencies according to C677T in patients.

Gene parameters	Genotype			Allele		*p*-value
	CC	CT	TT	C	T	0.08
Frequency (%)	26.70	41.88	31.42	47.64	52.36	

Data were presented as frequency. p-values were derived from Pearson’s chi-square test.

Based on the locus 677 genotype evaluation, the patients were divided into three groups: Control (homozygosity for the C allele), heterozygote mutated (heterozygosity), and mutated homozygote (homozygosity for the T allele). Age (*p* = 0.79), clinical conditions (BMI (*p* = 0.12), duration of infertility (*p* = 0.22), type of infertility (*p* = 0.96), and maternal baseline FSH concentration (*p* = 0.10) were not statistically different between the three groups (**Table 2**).

**Table 2:** Characteristics of the patients enrolled in this study.

Clinical characteristics	CC (*n* = 51)	CT (*n* = 80)	TT (*n* = 60)	*p*-value
Age of male (years; mean ± SD)	33.92 ± 6.49	33.19 ± 5.89	33.40 ± 5.96	0.79
Age of female spouse (years; mean ± SD)	32.56 ± 5.49	31.96 ± 5.58	32.17 ± 5.47	0.83
BMI of male (kg m^−2^; mean ± SD)	24.26 ± 3.08	23.81 ± 3.26	24.89 ± 2.87	0.12
BMI of female spouse (kg m^−2^; mean ± SD)	22.47 ± 3.18	22.59 ± 3.82	22.94 ± 2.98	0.73
Duration of infertility (years; mean ± SD)	4.05 ± 3.08	3.19 ± 2.50	3.38 ± 2.40	0.22
Primary infertility (*n* (%))	29 (56.86)	47 (58.75)	34 (56.67)	0.96
Secondary infertility (*n* (%))	22 (43.14)	33 (41.25)	26 (43.33)	
Maternal baseline FSH concentration (U/L; mean ± SD)	7.22 ± 1.60	7.11 ± 2.41	7.91 ± 2.52	0.10
Duration of female induction (days; mean ± SD)	9.78 ± 4.08	10.18 ± 3.75	9.97 ± 5.16	0.87
Female endometrial thickness on hCG day (mm; mean ± SD)	11.24 ± 2.62	11.12 ± 2.94	10.21 ± 2.90	0.09

BMI, body mass index; FSH, follicle stimulating hormone; hCG, human chorionic gonadotropin. Normally distributed quantitative. Variables were represented using mean ± SD. Qualitative variables were described as a percentage. Pearson’s chi-square test and analysis of variance (ANOVA) were employed for qualitative and quantitative variables with normal distribution.

There was also no statistically significant difference among the three groups in terms of semen parameters (including sperm concentration, morphology, motility, sperm DFI, and AsA level) (**Table 3**).

**Table 3:** Semen parameters of the study’s male population.

Sperm parameters	CC (*n* = 51)	CT (*n* = 80)	TT (*n* = 60)	*p*-value
Abstinence time (days; mean ± SD)	5.47 ± 4.77	5.88 ± 7.44	5.40 ± 3.96	0.87
Semen volume (ml; median (Q1, Q3))	2.90 (2.00, 3.80)	2.70 (1.90, 3.70)	2.75 (1.92, 3.87)	0.79
Sperm concentration (×10^6^ ml^−1^; median (Q1, Q3))	31.63 (10.95, 56.80)	36.75 (11.75, 97.69)	40.84 (8.71, 66.77)	0.39
Progressive motility (%; median (Q1, Q3))	16.84 (9.42, 33.00)	18.82 (12.66, 40.07)	20.92 (8.05, 29.77)	0.19
Total motility (%; median (Q1, Q3))	20.45 (12.42, 37.57)	25.28 (15.25, 44.71)	25.53 (10.43, 35.72)	0.18
Normal morphology (%; median (Q1, Q3))	3 (2, 4.5)	3 (1, 5)	3 (2, 4.75)	0.88
Leukocyte count (×10^6^ ml^−1^; median (Q1, Q3))	0.24 (0.08, 0.47)	0.16 (0.08, 0.34)	0.19 (0.08, 0.44)	0.50
AsA (%; median (Q1, Q3))	1 (0, 5)	0 (0, 2)	1 (0, 3.75)	0.06
DFI (%; mean ± SD)	22.68 ± 12.03 (*n* = 19)	22.97 ± 11.92 (*n* = 45)	21.59 ± 13.18 (*n* = 27)	0.89
HDS (%; mean ± SD)	11.41 ± 11.22 (*n* = 19)	9.37 ± 5.17 (*n* = 45)	8.85 ± 5.32 (*n* = 27)	0.43

AsA, antisperm antibody; DFI, DNA fragmentation index; HDS, high DNA stainability; Q1, 25th percentile; Q3, 75th percentile. p-values derived from analysis of variance (ANOVA) or the Kruskal–Wallis test for continuous variables.

### Correlations of paternal MTHFR polymorphism (C677T) with embryonic quality and pregnancy outcomes

The embryonic quality following ICSI intervention was first compared among the three groups. Results shown in **Table 4** revealed no significant differences in the normal fertilization rate (80.14% vs. 83.06% vs. 85.10%; *p* = 0.37), high-quality embryo rate (45.26% vs. 43.69% vs. 46.04%; (*p* = 0.72)), blastocyst formation rate (42.47% vs. 43.18% vs.39.38%; *p* = 0.62), implantation rate (42.47% vs. 36.25% vs. 41.22%; *p* = 0.62), and clinical pregnancy rate (64.71% vs. 58.75% vs. 66.67%; *p* = 0.59). We further examined whether any of the genotypes could influence the pregnancy outcomes, and the results showed that there were also no significant differences in terms of miscarriage rate (24.24% vs. 12.77% vs. 22.5%; *p* = 0.35)) or live birth rate (49.02% vs. 51.25% vs. 51.66%; (*p* = 0.96)) among the three groups (**Table 4**).

**Table 4:** Embryo quality and pregnancy outcome in association with MTHFR polymorphism.

Embryo and pregnancy parameters	CC (*n* = 51)	CT (*n* = 80)	TT (*n* = 60)	*p*-value
Number of MII oocytes (mean ± SD)	9.04 ± 4.97	9.70 ± 6.08	8.17 ± 6.10	0.31
Cleavage rate (*n* (%))	327/342 (95.61)	652/674 (96.74)	417/434 (96.08)	0.65
Normal fertilization rate (*n* (%))	327/408 (80.14)	652/785 (83.06)	417/490 (85.10)	0.37
High-quality embryo rate (*n* (%))	148/327 (45.26)	343/785 (43.69)	192/417 (46.04)	0.72
Blastocyst formation rate (*n* (%))	79/186 (42.47)	171/396 (43.18)	102/259 (39.38)	0.62
Implantation rate (*n* (%))	44/107 (42.47)	58/160 (36.25)	47/114 (41.22)	0.62
Clinical pregnancy rate (*n* (%))	33/51 (64.71)	47/80 (58.75)	40/60 (66.67)	0.59
Miscarriage rate (*n* (%))	8/33 (24.24)	6/47 (12.77)	9/40 (22.5)	0.35
Live birth rate (*n* (%))	25/51 (49.02)	41/80 (51.25)	31/60 (51.66)	0.96
Singleton birth rate (*n* (%))	19/25 (76)	38/41 (92.68)	28/31 (90.32)	0.17
Multiple-birth rate (*n* (%))	6/25 (24)	3/41 (7.32)	3/31 (9.68)	

p-values derived from Pearson’s chi-square test or Fisher’s exact test and ANOVA.

### Association of paternal MTHFR polymorphism (C677T) with neonatal outcome

The neonatal outcome was evaluated based on gestational age at delivery, preterm delivery, birth height, birth weight, neonatal sex, and Apgar score among the three groups (**Table 5**). Our results showed that paternal MTHFR polymorphism may not contribute to the neonatal outcome, with no significant difference found in terms of preterm delivery rate (24% vs. 14.63% vs. 9.67%; *p* = 0.35), birth height (*p* = 0.75), birth weight (*p* = 0.35), neonatal sex (*p* = 0.48), gestational age at delivery (*p* = 0.24), and Apgar score (*p* = 0.34). In addition, no statistically significant differences in terms of birth defects (0% vs. 2% vs. 9%; *p* = 0.23) were observed among the three MTHFR C677T genotype groups. The birth defect of one case in the CT group is congenital heart disease, and the birth defect of three cases in the TT group is of patent foramen ovale of heart, congenital heart disease, and cerebral hemangioma.

**Table 5:** Neonatal outcomes in association with MTHFR polymorphism.

Neonatal birth parameters	CC (*n* = 51)	CT (*n* = 80)	TT (*n* = 60)	*p*-value
Gestational age (weeks; mean ± SD)	38.33 ± 2.26	39.18 ± 2.41	38.23 ± 3.07	0.24
Preterm delivery rate (*n* (%))	6/25 (24)	6/41 (14.63)	3/31 (9.67)	0.35
Cesarean delivery rate (*n* (%))	19/25 (76)	29/41 (71)	23/31 (74)	0.89
Normal delivery rate (*n* (%))	6/25 (24)	12/41 (29)	8/31 (26)	
Female baby birth rate (*n* (%))	19/31 (61.29)	22/44 (50)	16/34 (47.05)	0.48
Male baby birth rate (*n* (%))	12/31 (38.71)	22/44 (50)	18/34 (52.95)	
Apgar score (mean ± SD)	9.90 ± 0.30	9.93 ± 0.26	9.76 ± 0.07	0.34
Birth height (cm; mean ± SD)	48.61 ± 2.73	49.14 ± 2.89	49.18 ± 4.33	0.75
Birth weight (kg; mean ± SD)	3.01 ± 0.69	3.25 ± 0.70	3.16 ± 0.72	0.35
Neonatal birth abnormality rate (*n* (%))	0/31 (0)	1/44 (2)	3/34 (9)	0.23

p-values derived from Pearson’s chi-square test or Fisher’s exact test and ANOVA.

## Discussion

The MTHFR enzyme is a key enzyme in folate metabolism and is necessary for the homocysteine conversion to methionine (22). The 677 C>T MTHFR polymorphism results in decreased activity of MTHFR, causing an increase in homocysteine (Hcy) concentrations in body fluids (12). DNA damage and incorrect methylation caused by excess Hcy can impact the developing gametes and embryos (23). There has been a long-standing interest in the relationship between MTHFR polymorphism(s) and pregnancy outcomes (16). Likewise, numerous studies have investigated the association between maternal MTHFR genetic polymorphism and adverse health outcomes (11, 12, 19). According to several reports, MTHFR polymorphisms in 677 C>T and hyperhomocysteinemia lead to decreased global sperm DNA methylation and are considered to be risk factors for semen parameters and human infertility (7, 15). Testing for paternal MTHFR polymorphisms may also be crucial because it is widely acknowledged that the paternal genome has a significant role in embryonic development (24). Nevertheless, the correlation between MTHFR genetic polymorphism variants in men and clinical outcomes has not been well established. The present study is the first to investigate the effects of paternal MTHFR polymorphisms on clinical outcomes following ICSI treatment in terms of embryonic quality, pregnancy, and neonatal outcomes. Based on our findings, the male MTHFR genetic polymorphism (677 C>T) does not affect embryo quality, the frequency of early losses, or neonatal outcomes after ICSI treatment. Dobson et al. previously found that paternal MTHFR C677T was not associated with pregnancy rate, positive pregnancy tests, and clinical pregnancy rate in IVF treatment (20). Meanwhile, Poorang et al. also indicated that no significant difference was observed in the frequency of the methylated MTHFR epigenotype between recurrent pregnancy loss (RPL) and non-RPL men (15). This is somewhat surprising; one possible reason for the lack of association between paternal MTHFR genotype and ICSI outcomes in our study is that oocytes may store folic acid or other methyl donors, such as SAM. It is known to all that according to the Chinese guidelines for the prevention of neural tube defects (NTDs) by periconceptional folic acid supplementation, all women trying to conceive ought to take 400 or 800 µg of folate daily at the time of preparation (25). Another possible reason is that the sample size of ICSI patients (*N* = 191) in our research seems insufficient. This is one of the limitations of our study. It should be emphasized that although there was no statistically significant difference, the proportion of birth abnormalities was higher in the MTHFR 677TT group. Possibly, increasing the sample size could bring about more convincing results.

There has recently been evidence that the MTHFR polymorphism (677 C>T) is associated with male infertility in different populations (8, 13, 14, 26). Indeed, scientists found the 222 Val allele (677CT) and Val–Val (677TT) genotypes were significantly more frequent in azoospermic and oligozoospermic men (27–29). A similar result was found in our study of male patients with severe and very severe oligozoospermia. In our study, however, we found that sperm DFI was not affected by the MTHFR polymorphism, consistent with several reports published by Cornet et al. (7).

In conclusion, we did not find any association between the paternal MTHFR C677T polymorphisms and embryonic quality, pregnancy outcomes, including miscarriage, live birth rate, or neonatal outcomes, such as preterm delivery, birth height, birth weight, or gestational age at delivery. Further studies investigating the combined effect of paternal MTHFR polymorphisms on ICSI outcomes with larger sample sizes are recommended.

## Data availability statement

The original contributions presented in the study are included in the article/supplementary material. Further inquiries can be directed to the corresponding authors.

## Ethics statement

The studies involving human participants were reviewed and approved by the Ethical Committee of the First Affiliated Hospital of USTC (Approval ID: 2022-RE-261). The patients/participants provided their written informed consent to participate in this study.

## Author contributions

JH and YW designed the studies. YW and BX collected the data. XJ, YW, and WL performed the data analysis. JH and XJ wrote the manuscript. BX revised the manuscript. All authors reviewed and approved the final manuscript.

## References

[1] Mani S Ghosh J Coutifaris C Sapienza C Mainigi M . Epigenetic changes and assisted reproductive technologies. Epigenetics (2020) 15:12–25. doi: 10.1080/15592294.2019.1646572 31328632 PMC6961665

[2] Gleicher N Barad DH . Assessing in-vitro fertilisation at age 40 years. Lancet (2019) 23:1181–3. doi: 10.1016/S0140-6736(18)32070-1. 30655016

[3] Rubino P Vigano P Luddi A Piomboni P . The ICSI procedure from past to future: a systematic review of the more controversial aspects. Hum Reprod Update (2016) 22:194–227. doi: 10.1093/humupd/dmv050 26586241

[4] Moubasher A Taha EA Elnashar EM Abdel Maged AAA Zahran AM Sayed HH . Semen parameters on the intracytoplasmic sperm injection day: Predictive values and cutoff thresholds of success. Clin Exp Reprod Med (2021) 48:61–8. doi: 10.5653/cerm.2020.03965 PMC7943354 33648046

[5] Pereira R Oliveira J Ferraz L Barros A Santos R Sousa M . Mutation analysis in patients with total sperm immotility. J Assisted Reprod Genet (2015) 32:893–902. doi: 10.1007/s10815-015-0474-6 PMC4491072 25877373

[6] Santos F Hyslop L Stojkovic P Leary C Murdoch A Reik W . Evaluation of epigenetic marks in human embryos derived from IVF and ICSI. Hum Reprod (2010) 25:2387–95. doi: 10.1093/humrep/deq151 20634187

[7] Cornet D Cohen M Clement A Amar E Fournols L Clement P . Association between the MTHFR-C677T isoform and structure of sperm DNA. J Assisted Reprod Genet (2017) 34:1283–8. doi: 10.1007/s10815-017-1015-2 PMC5633564 28842818

[8] Aliakbari F Pouresmaeili F Eshghifar N Zolghadr Z Azizi F . Association of the MTHFR 677C>T and 1298A>C polymorphisms and male infertility risk: a meta-analysis. Reprod Biol Endocrinol RB&E (2020) 18:93. doi: 10.1186/s12958-020-00649-1 32912251 PMC7488080

[9] Meng H Huang S Yang Y He X Fei L Xing Y . Association between MTHFR polymorphisms and the risk of essential hypertension: An updated meta-analysis. Front Genet (2021) 12:698590. doi: 10.3389/fgene.2021.698590 34899823 PMC8662810

[10] Karahan G Chan D Shirane K McClatchie T Janssen S Baltz JM . Paternal MTHFR deficiency leads to hypomethylation of young retrotransposons and reproductive decline across two successive generations. Development (2021) 148 (13): dev199492. doi: 10.1242/dev.199492 34128976 PMC8276981

[11] Shahrokhi SZ Kazerouni F Ghaffari F Rahimipour A Omrani MD Arabipoor A . The relationship between the MTHFR C677T genotypes to serum anti-mullerian hormone concentrations and *In vitro* Fertilization/Intracytoplasmic sperm injection outcome. Clin Lab (2017) 63:927–34. doi: 10.7754/Clin.Lab.2016.161104 28627828

[12] Ye F Zhang S Qi Q Zhou J Du Y Wang L . Association of MTHFR 677C>T polymorphism with pregnancy outcomes in IVF/ICSI-ET recipients with adequate synthetic folic acid supplementation. Biosci Trends (2022) 16:282–90. doi: 10.5582/bst.2021.01306 35691911

[13] Gong M Dong W He T Shi Z Huang G Ren R . MTHFR 677C>T polymorphism increases the male infertility risk: a meta-analysis involving 26 studies. PloS One (2015) 10:e0121147. doi: 10.1371/journal.pone.0121147 25793386 PMC4368707

[14] Wei B Xu Z Ruan J Zhu M Jin K Zhou D . MTHFR 677C>T and 1298A>C polymorphisms and male infertility risk: a meta-analysis. Mol Biol Rep (2012) 39:1997–2002. doi: 10.1007/s11033-011-0946-4 21643754

[15] Poorang S Abdollahi S Anvar Z Tabei SMB Jahromi BN Moein-Vaziri N . The impact of methylenetetrahydrofolate reductase (MTHFR) sperm methylation and variants on semen parameters and the chance of recurrent pregnancy loss in the couple. Clin Lab (2018) 64:1121–8. doi: 10.7754/Clin.Lab.2018.171231 30146842

[16] Dai C Fei Y Li J Shi Y Yang X . A novel review of homocysteine and pregnancy complications. BioMed Res Int (2021) 2021):6652231. doi: 10.1155/2021/6652231 34036101 PMC8121575

[17] Zhu Y Wu T Ye L Li G Zeng Y Zhang Y . Prevalent genotypes of methylenetetrahydrofolate reductase (MTHFR) in recurrent miscarriage and recurrent implantation failure. J Assisted Reprod Genet (2018) 35:1437–42. doi: 10.1007/s10815-018-1205-6 PMC6086799 29785531

[18] Chen L Chen H Wang X Wei B Wu Z Chen S . Association of homocysteine with IVF/ICSI outcomes stratified by MTHFR C677T polymorphisms: a prospective cohort study. Reprod Biomed Online (2021) 43:52–61. doi: 10.1016/j.rbmo.2021.04.009 34016520

[19] D'Elia PQ dos Santos AA Bianco B Barbosa CP Christofolini DM Aoki T . MTHFR polymorphisms C677T and A1298C and associations with IVF outcomes in Brazilian women. Reprod Biomed Online (2014) 28:733–8. doi: 10.1016/j.rbmo.2014.02.005 24746944

[20] Dobson AT Davis RM Rosen MP Shen S Rinaudo PF Chan J . Methylenetetrahydrofolate reductase C677T and A1298C variants do not affect ongoing pregnancy rates following IVF. Hum Reprod (2007) 22:450–6. doi: 10.1093/humrep/del396 17053001

[21] Hua J Guo L Yao Y Hu W Wan YY Xu B . Biallelic mutations in WDR12 are associated with male infertility with tapered-head sperm. Asian J Androl (2022). doi: 10.4103/aja202269 PMC10226492 36178131

[22] Rotondo JC Lanzillotti C Mazziotta C Tognon M Martini F . Epigenetics of Male infertility: The role of DNA methylation. Front Cell Dev Biol (2021) 9:689624. doi: 10.3389/fcell.2021.689624 34368137 PMC8339558

[23] Liu L Lin Z Lin P Jiang Z . Association between serum homocysteine level and unexplained infertility in in vitro fertilization/intracytoplasmic sperm injection (IVF/ICSI): A retrospective, hospital-based, case-control study. J Clin Lab Anal (2020) 34:e23167. doi: 10.1002/jcla.23167 31876071 PMC7246389

[24] Tarozzi N Nadalini M Coticchio G Zaca C Lagalla C Borini A . The paternal toolbox for embryo development and health. Mol Hum Reprod (2021) 27. doi: 10.1093/molehr/gaab042 34191013

[25] Liu J Li Z Ye R Ren A Liu J . Folic acid supplementation and risk for congenital limb reduction defects in China. Int J Epidemiol (2019) 48:2010–7. doi: 10.1093/ije/dyz130 31257442

[26] Mfady DS Sadiq MF Khabour OF Fararjeh AS Abu-Awad A Khader Y . Associations of variants in MTHFR and MTRR genes with male infertility in the Jordanian population. Gene (2014) 536:40–4. doi: 10.1016/j.gene.2013.12.001 24334125

[27] Gurkan H Tozkir H Goncu E Ulusal S Yazar M . The relationship between methylenetetrahydrofolate reductase c.677TT genotype and oligozoospermia in infertile male patients living in the trakya region of Turkey. Andrologia (2015) 47:1068–74. doi: 10.1111/and.12380 25428700

[28] Chellat D Rezgoune ML Hamane D Semmame O Benlatreche C Abadi N . Influence of methylenetetrahydrofolate reductase C677T gene polymorphisms in Algerian infertile men with azoospermia or severe oligozoospermia. Genet Testing Mol Biomarkers (2012) 16:874–8. doi: 10.1089/gtmb.2011.0367 22928696

[29] Gava MM Chagas Ede O Bianco B Christofolini DM Pompeo AC Glina S . Methylenetetrahydrofolate reductase polymorphisms are related to male infertility in Brazilian men. Genet Testing Mol Biomarkers (2011) 15:153–7. doi: 10.1089/gtmb.2010.0128 21138341

